# The timing of testing influences skill retention after basic life support training: a prospective quasi-experimental study

**DOI:** 10.1186/s12909-019-1881-7

**Published:** 2019-12-04

**Authors:** Enikő Kovács, Zsigmond Máté Jenei, Katalin Csordás, Gábor Fritúz, Balázs Hauser, V. Anna Gyarmathy, Endre Zima, János Gál

**Affiliations:** 1 Department of Anaesthesiology and Intensive Therapy, Semmelweis University, P.O.B. 2, Budapest, H-1428 Hungary; 2 3rd Department of Internal Medicine, Semmelweis University, P.O.B. 2, Budapest, H-1428 Hungary; 3 National Institute of Hematology and Infectious Diseases, Central Hospital of Southern Pest, Albert Flórián út 5-7, Budapest, H-1097 Hungary; 4 Johns Hopkins University, Baltimore, MD USA; 5 Heart and Vascular Center, Semmelweis University, P.O.B. 2, Budapest, H-1428 Hungary

**Keywords:** Cardiopulmonary resuscitation, Basic life support, Out-of-hospital cardiac arrest, Skill retention, Testing effect, Exam

## Abstract

**Background:**

Proper basic life support (BLS) is key in improving the survival of out-of-hospital cardiac arrest. BLS skills deteriorate in three to 6 months after training. One method to improve skill retention may be using the “testing effect” to test skills at the end of a BLS course. The aim of our study was to investigate whether either testing or the timing of such testing after BLS training have any influence on skill retention.

**Methods:**

This was a post-test only, partial coverage, prospective quasi-experimental study designed to evaluate a BLS training course among 464 fifth year medical students at Semmelweis University in the first semester of 2013/2014. Groups were systematically but non-randomly assigned to either a control group that took no exam or one of two experimental groups that took an exam (*N* = 179, NoExam group; *N* = 165, EndExam group – exam at the end of the BLS training; *N* = 120, 3mExam group – exam 3 months after the BLS training). The ability to perform ten prescribed essential BLS steps was evaluated during a skill retention assessment 2 months after the course in the NoExam, 2 months after the course (and the exam) in the EndExam and 5 months after the course (2 months after the exam) in the 3mExam group to measure skill retention and the effect of our intervention. Scores were calculated for each BLS step, and also summed up as a total score. We used Kruskal-Wallis test to assess differences in skill retention.

**Results:**

Overall, NoExam and EndExam groups showed similar skill retention. The mean total score (and many of the sub-scores) of students was significantly higher in the 3mExam group compared to both the NoExam and the EndExam groups, and there was no difference in the total score (and many of the sub-scores) of the latter two groups. The 3mExam group had less variability in total scores (and many of the sub-scores) than the other two groups.

**Conclusion:**

Our study provides evidence that testing these skills 3 months after BLS training may be more effective than either testing immediately at the end of the course or no testing at all.

## Background

Sudden cardiac arrest is still one of the leading causes of death in Europe and the United States [1]. Performing immediate and proper cardiopulmonary resuscitation (CPR) may increase survival [2]. However, teaching simple and complex technical skills, which need not only theoretical knowledge but also a development in psychomotor abilities, is a challenge in several fields of medical education. One of the most investigated specialties in skill teaching is emergency medicine, because providers’ performance has influence on the patients’ outcome in this field [3].

The ability to perform effective basic life support (BLS) is crucial for every healthcare worker and a valuable skill for laypeople as well. After training, BLS skills deteriorate in three to 6 months if not used [4–6]. Numerous studies have investigated how to improve BLS skill retention and sought to identify the best educational method to achieve long term BLS skill retention. Refresher courses [7, 8], special feedback devices [9], the use of a virtual patient [10], and beginning BLS education during childhood [11] have all been shown to improve BLS skill retention. Simulation based learning has also been shown to be effective, but it is expensive, time consuming and needs a minimum number of human resources to secure effectiveness [12, 13].

Testing skills after BLS training might be a simple, cheap, and time-efficient method of prolonging skill retention. Testing CPR skills at the end of a CPR course increases learning outcome [14]. Medical students who took an exam at the end of BLS training had better skill retention assessed 2 weeks after the course compared to using the same time period for practicing CPR [15]. Skill retention after such testing may last at least for 6 months [16]. A randomised non-inferiority trial also showed the potential of repetitive sessions of formative self-testing to refresh CPR skills [17]. The terminology “testing effect”, which refers to the fact that repeated retrieval of memories during testing better enhances knowledge retention than repeated studying [18], might be the basis for this finding. Previous studies have shown that the testing effect occurs even if there is no feedback after the exam [19].

However, only a few studies have investigated the testing effect with respect to BLS skill retention [14–17]. To our knowledge, no studies have investigated whether the timing of testing influences learning outcome. Therefore, the aim of our study was to investigate the influence of testing and the timing of the examination on BLS skill retention.

## Methods

### Study design and participants

This was a post-test only, partial coverage, prospective quasi-experimental study designed to evaluate a BLS training course among 464 fifth year medical students at Semmelweis University in the first semester of academic year 2013/2014. Students were participants of a compulsory Emergency Medicine course, which contained thirteen 70-min long lecture sessions (a lecture session covered several topics) and five 90-min long practice sessions during the study period (the BLS training formed a part of this course). They supposedly had had no organized BLS training until this course, with the exception of some basic first aid training that was part of their curriculum in the first year of their medical studies.

The Semmelweis University Regional and Institutional Committee of Science and Research Ethics approved our study. The informed consent was waived due to the nature of the study based on our national regulations. Participation in the study and study results did not affect students’ grades and the results were processed anonymously.

### Group assignment

**Fig. 1:**
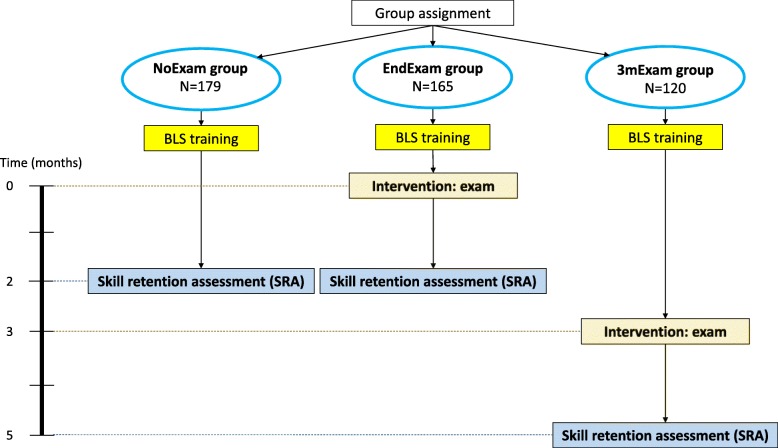
Group assignment and the diagram of study design

Since the Study and Examination Policy of our university did not allow us to perform a randomized controlled study among students for this registered course, our study employed a post-test only, partial coverage, prospective quasi-experimental design. Groups were systematically but non-randomly assigned to either a control group or one of two experimental groups based on consecutive sampling according to their date of participation in the BLS practice session: the first 12 groups (*N* = 179 students) were assigned into the NoExam control group, the second 12 groups (*N* = 165 students) were assigned into the EndExam experimental group and the third 9 groups (*N* = 120 participants) were assigned into the 3mExam experimental group (Fig. 1). Participation in the groups was blinded in a way that all participants at the beginning of the course thought they were not going to have an exam, and those who were taking exams were told during their training session.

### BLS training

As part of this study, participants received a 45-min BLS lecture and a 90-min BLS training session according to the European Resuscitation Council (ERC) Guidelines 2010 [20]. Working in pairs, the students had to solve simple BLS scenarios after a four-step presentation; one student performed BLS, and the other helped him/her. Everyone performed at least one complete BLS algorithm. Each student received the same education and was presented with the same scenario.

Peyton’s Four-step Approach [21] was used as the teaching method during the training sessions, which contains four steps:

- Step 1: Real-time demonstration on the manikin - the instructor shows the process of BLS without any comments.
- Step 2: Repeated demonstration on the manikin with explanation – the instructor displays the process again slowly and explains it in detail.
- Step 3: Demonstration lead by a student on the manikin – the instructor shows the process of BLS again based on the instructions of a selected student.
- Step 4: Demonstration performed by a student on the manikin – the student who gave the instructions in the previous step performs BLS under the inspection of the instructor.

The teacher to student ratio during the training sessions was 1:7. Ambu Man C Torso® (Ambu A/S, Copenhagen, Denmark) manikins were used during the BLS simulation training and exams. Students were observed and corrected by continuous assessment and finished the training only when they have performed a satisfactory BLS technique.

### Intervention – practical exam (“testing effect”)

As it can further be seen in Fig. 1, the NoExam group had no practical exam after the training. Students in the EndExam group took a practical exam immediately following their BLS course, and students in the 3mExam group took the practical exam 3 months after the BLS course. The three-month period for the exam for the third group was chosen because it mirrors the official end-semester examination period. As a note, students in the 3mExam group had no organized opportunity to practice BLS between their course and their exam and were specifically asked not to train during these 3 months.

### Evaluation – skill retention assessment (SRA)

The evaluation consisted of a skill retention assessment (SRA), which was identical in nature with the exam, meaning that participants knew that they were being assessed and they were assessed using identical criteria, but during the exam they were told that their assessments were scored, while during the SRA they were not told that their assessments were scored.

As part of the evaluation, students had to enact and resolve a BLS scenario, supervised by independent ERC instructors who had not been involved in the training. The following ten BLS steps were tested: 1. shouting for help, 2. examining consciousness, 3. testing vital signs, 4. call for advanced life support (ALS) team, 5. position of hands on the chest, 6. depth of chest compressions, 7. rate of chest compressions, 8. consistency of chest compressions, 9. maintaining a 30:2 compression to ventilation ratio, and 10. duty cycle.

A checklist indicating a step correct vs. incorrect was used for evaluation. The chest compression depth was measured by the built-in sensor in the Ambu Man Torso®, and chest compression frequency was determined using a stopwatch. A BLS step was considered correct if it met the ERC guidelines [20] described above, and if it was performed correctly at least 75% of the time during the assessment. We recorded the correctly and incorrectly performed events. The students received a score of 1 for a properly implemented step and a score of 0 for an incorrect performance, which was recorded in the evaluation sheet.

As Fig. 1 shows, the SRA took place 2 months after the BLS training for the NoExam and EndExam groups and 5 months after the BLS training (i.e., 2 months after the practical exam) for the 3mExam group. The two-month period for the follow-up assessment (SRA) was chosen because it was the longest time period in which all three groups were able to complete the evaluation of the course within the academic year.

### Statistical analysis

A summary score was calculated by adding up the individual BLS scores in the SRA. The distribution of the average scores for each BLS step and for the total score was compared across the three groups using the Kruskal-Wallis test overall and the Dunn post-hoc test across groups. The level of significance was set at *p* < 0.05. Statistical analysis was performed using SPSS v13.0 (SPSS Inc., Chicago, IL). Figures were created using GraphPad Prism version 8.1.1. (GraphPad Software, La Jolla, CA).

## Results

**Fig. 2:**
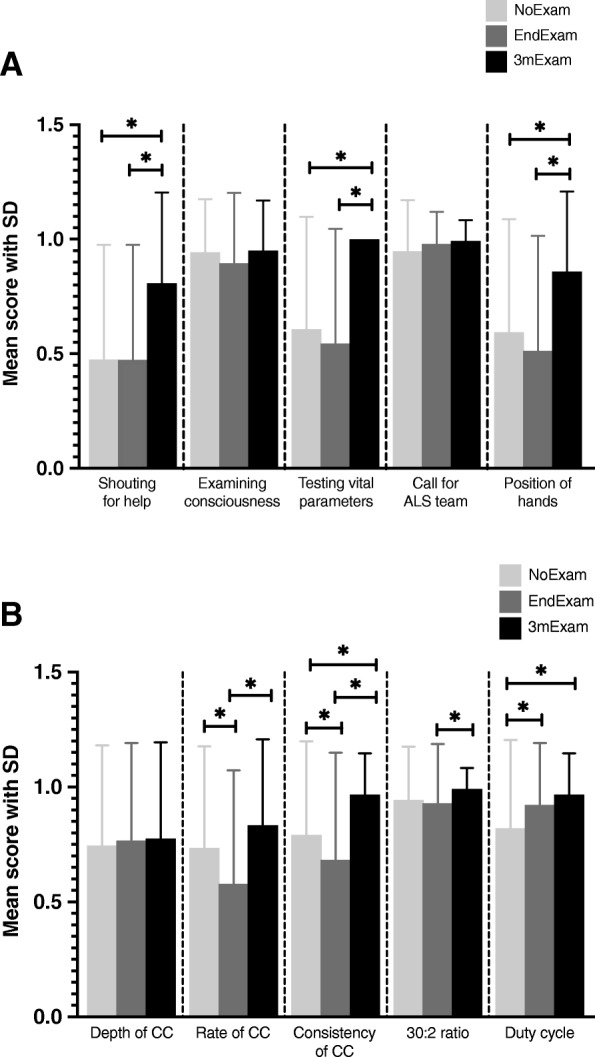
**a** and **b** A comparison of BLS step mean scores by group using Kruskal-Wallis test and post-hoc analysis. Students in 3mExam group showed the best skill retention during the final evaluation. NoExam and EndExam groups’ performance was similar, except rate and consistency of chest compression, and duty cycle. Scoring based on 0 = incorrect and 1 = correct. Significant differences (*p* < 0.05) and the results of post-hoc analysis are marked with a star. CC: chest compression, SD: standard deviation

**Fig. 3:**
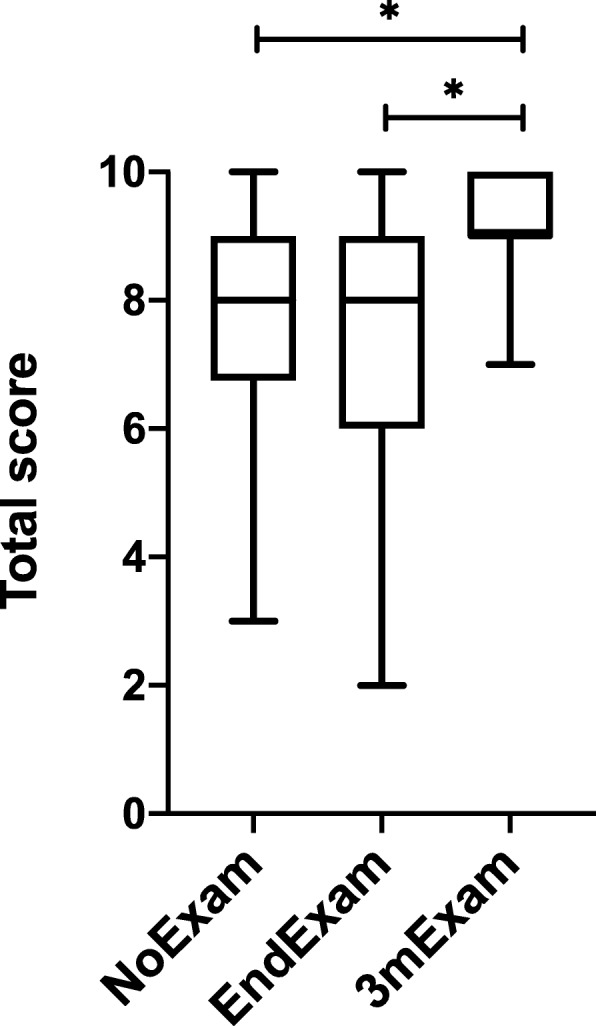
A comparison of total scores by group using Kruskal-Wallis test and post-hoc analysis. 3mExam group’s total score was significantly higher than the total score reached by NoExam or EndExam groups. The total score is a sum of the individual BLS scores. Significant differences (*p* < 0.05) and the results of post-hoc analysis are marked with a star. Box-and-whiskers plot: the box extends from the 25 to 75 percentile and interprets the mean value, while the whiskers show minimum and maximum values

The gender distribution was not significantly different (*p* = 0.228) between the groups (NoExam: 61%, EndExam: 52%, 3mExam: 58% females – data not shown in table or figure). As can be seen in Table 1, the SRA 2 months after the training in the NoExam, 2 months after the training and the exam in the EndExam, and 2 months after the exam in the 3mExam group showed significantly different scores across the groups regarding shouting for help, testing vital signs, position of hands, rate of chest compression, consistency of chest compression, 30:2 ratio, duty cycle, and total score. There was no significant difference in examining consciousness, calling for ALS team, and depth of chest compression. Furthermore, as can be seen in Fig. 2a and b showing the post-hoc differences across groups, students in the 3mExam group performed significantly better than students in either the NoExam or the EndExam groups in shouting for help, testing vital signs, positioning of hands, and consistency of chest compression. In addition, the 3mExam group had a significantly better performance in rate of chest compression compared to the EndExam group (which had significantly lower scores for this step than the NoExam group), as well as in keeping the 30:2 ratio. Duty cycle was retained significantly better in the 3mExam group compared to the NoExam group. The NoExam group had a higher mean score in rate and consistency of chest compressions than the EndExam group, however the EndExam group’s skill retention was significantly better in duty cycle compared to the NoExam group. Overall, the NoExam and the EndExam groups showed similar skill retention. The mean total score of students was significantly higher in the 3mExam group compared to both the NoExam and the EndExam groups, and there was no difference in the total score of the latter two groups (Fig. 3). Moreover, the 3mExam group had less variability in total scores (and many of the sub-scores) than the other two groups, and the minimum total score for the 3mExam group was only 1 point lower than the average of the other two groups.

**Table 1:** A comparison of BLS step mean scores and total score by group using Kruskal-Wallis test

	NoExam *N* = 179	EndExam *N* = 165	3mExam *N* = 120	
BLS step	Mean score ± SD			*p*
Shouting for help	0.5 ± 0.5	0.5 ± 0.5	0.8 ± 0.4	**< 0.001**
Examining consciousness	0.9 ± 0.2	0.9 ± 0.3	1.0 ± 0.2	0.143
Testing vital signs	0.6 ± 0.5	0.5 ± 0.5	1.0 ± 0.0	**< 0.001**
Call for ALS team	0.9 ± 0.2	1.0 ± 0.1	1.0 ± 0.1	0.063
Position of hands	0.6 ± 0.5	0.5 ± 0.5	0.9 ± 0.4	**< 0.001**
Depth of chest compression	0.7 ± 0.4	0.8 ± 0.4	0.8 ± 0.4	0.812
Rate of chest compression	0.7 ± 0.4	0.6 ± 0.5	0.8 ± 0.4	**< 0.001**
Consistency of chest compression	0.8 ± 0.4	0.7 ± 0.5	1.0 ± 0.2	**< 0.001**
30:2 ratio	0.9 ± 0.2	0.9 ± 0.3	1.0 ± 0.1	**0.046**
Duty cycle	0.8 ± 0.4	0.9 ± 0.3	1.0 ± 0.2	**< 0.001**
Total score (0–10)	7.6 ± 1.6	7.3 ± 1.8	9.1 ± 0.8	**< 0.001**

Scoring based on 0 = incorrect and 1 = correct. The total score is a sum of the individual BLS scores. *SD* Standard deviation

## Discussion

Improving skill retention in BLS education is an important issue because it may lead to a higher success rate of resuscitation and improve outcome [3, 22]. Quality of chest compressions is one of the most important factors that determines the outcome of a cardiac arrest patient [20]. It has been shown that formal certified courses and their periodic renewal improve the outcome of resuscitation [23]. However, a significant degree of skill decay can occur within three to 6 months after training [4–6]. Several methods have been investigated as a tool to prolong skill retention [7–11]. Our goal was to find an effective skill retention method that is simple and time- and cost-effective. We used simulation training during our courses and exams, because it is of great benefit for the students and a proper teaching tool of BLS skills [24].

Our results demonstrate that testing and the timing of testing after BLS training do influence BLS skill retention among senior medical students. We found that students who took an exam 3 months after their BLS training had significantly better overall skill retention assessed during the SRA 2 months after the exam than students who either took no exam or had the exam immediately after the training.

One may wonder why we chose 3 months after training as the time point for giving the BLS skills exam to students in the third group. The timing means that for students in the 3mExam group, 5 months elapsed between the end of their BLS training and the skill retention assessment, compared with 2 months for the other two groups. We chose this time point because it mirrors the official end-semester examination period. In addition, for each group, we assessed BLS skill retention 2 months after the last educational intervention, as the exam was considered as an intervention due to the “testing effect”.

During the exams and the SRA, we evaluated ten essential BLS components, which may contribute to the detection of cardiac arrest and a successful resuscitation and therefore influence patient outcome. These steps are important in recognizing cardiac arrest (examining consciousness, testing vital parameters), calling for help, and performing correct chest compressions (position of hands, depth, frequency and consistency of chest compressions, maintaining 30:2 compression to ventilation ratio, and duty cycle). It is well known that early recognition and immediate CPR may double or triple the likelihood of survival of in- and out-of-hospital cardiac arrest with ventricular fibrillation [25, 26]. There is also evidence that the proper rate and depth of compression increase the rate of return of spontaneous circulation [27, 28].

It has already been shown that testing skills after BLS training improves skill retention more than spending the same duration with additional training at the end of a course [15]. This finding may be the result of the “testing effect”, a phenomenon based on the fact that retrieval of memories during a test is more effective in creating long-term memory than additional study and training time [15, 16, 29]. From a psychological point of view, the stress response might play an important role in improving skill retention, and remembrance during testing acts as a stress factor. One of the neuronal changes that occurs in response to stress plays an important role in creating memories [30]. It has also been shown that prior knowledge of testing improves sensorimotor learning [31]. Students in the EndExam group became aware of testing only at the beginning of the training, which might have influenced their performance negatively in some sensorimotor skills. Students in the 3mExam group had more time to prepare psychologically for their exam and therefore had a longer exposure to the stress effect. However, they did not get organized re-trainings to practice BLS skills before their exam. We also need to consider that testing 3 months after training may have a more complex educational impact and it should not be taken as a single testing step.

Surprisingly we found no significant difference between NoExam and EndExam groups, which contradicts formerly published data [14–16]. As we mentioned previously, it may mirror the complexity of stress response. These results highlight the fact that further investigations are needed to understand the effectiveness of testing and timing of testing after BLS training.

### Limitations

Some limitations of our study need to be considered. Although our instructors received the same training in teaching BLS and performed the same quality teaching based on the standard ERC instructors’ guidelines, it would have been preferable if the same instructor had taught all of the students. The exams were also administered by multiple instructors. Although we tried to evaluate the students’ performances using standard guidelines, we cannot rule out teacher-related differences [32]. We also need to highlight the lack of information about the preparation of the 3mExam group for their exam. They did not have an organized opportunity to practice after the course, but we cannot rule out that some might have practiced their skills in some other training format.

## Conclusions

Properly taught BLS skills deteriorate in three to 6 months. One of the methods to improve skill retention is to test these skills after the training, though the proper timing of testing is unknown. We investigated in our prospective quasi-experimental study the effect of testing and the timing of the examination on BLS skill retention after a BLS course. We found that the timing of testing influences skill retention in fifth year medical students. Our study provides evidence that testing these skills 3 months after training may be more effective than either testing immediately at the end of the course or not testing at all.

## Data Availability

All data generated and analysed during this study are available from the corresponding author on reasonable request.

## Abbreviations

ALS: Advanced life support
BLS: Basic life support
CPR: Cardiopulmonary resuscitation
ERC: European Resuscitation Council
SRA: Skill retention assessment
